# Evaluation of Two Methods to Concentrate SARS-CoV-2 from Untreated Wastewater

**DOI:** 10.3390/pathogens10020195

**Published:** 2021-02-12

**Authors:** Roger Dumke, Magali de la Cruz Barron, Reinhard Oertel, Björn Helm, Rene Kallies, Thomas U. Berendonk, Alexander Dalpke

**Affiliations:** 1 Institute of Medical Microbiology and Virology, Technische Universität Dresden, Fetscherstrasse 74, 01307 Dresden, Germany; Alexander.Dalpke@uniklinikum-dresden.de; 2 Institute of Hydrobiology, Technische Universität Dresden, Helmholtzstrasse 10, 01069 Dresden, Germany; magali.de_la_cruz_barron@tu-dresden.de (M.d.l.C.B.); thomas.berendonk@tu-dresden.de (T.U.B.); 3 Department of River Ecology, Helmholtz Centre for Environmental Research—UFZ, Brückstrasse 3a, 39114 Magdeburg, Germany; 4 Institute of Clinical Pharmacology, Technische Universität Dresden, Fetscherstrasse 74, 01307 Dresden, Germany; reinhard.oertel@tu-dresden.de; 5 Institute of Urban and Industrial Water Management, Technische Universität Dresden, Helmholtzstrasse 10, 01069 Dresden, Germany; bjoern.helm@tu-dresden.de; 6 Department of Environmental Microbiology, Helmholtz Centre for Environmental Research—UFZ, Permoserstrasse 15, 04318 Leipzig, Germany; rene.kallies@ufz.de

**Keywords:** SARS-CoV-2, wastewater, virus concentration, wastewater-based epidemiology, monitoring, surveillance

## Abstract

Use of wastewater-based epidemiology as a tool to record and manage the course of SARS-CoV-2 infections in human populations requires information about the efficiency of methods to concentrate the virus from wastewater. In the present study, we spiked untreated wastewater with quantified SARS-CoV-2 positive clinical material and enriched the virus by polyethylene glycol precipitation and ultrafiltration with Vivaspin 10 kDa MWCO columns. SARS-CoV-2 was detected and quantified by reverse transcription quantitative PCR (E- and S-gene) and droplet digital PCR. The concentration of virus with precipitation resulted in mean recoveries between 59.4% and 63.7% whereas rates from 33.0% to 42.6% after ultrafiltration of samples were demonstrated. The results suggest that the use of both methods allows an effective and practicable enrichment of SARS-CoV-2 from raw wastewater.

## 1. Introduction

In December 2019, the severe acute respiratory syndrome coronavirus 2 (SARS-CoV-2) firstly emerged in Wuhan, China. In a relatively short time, SARS-CoV-2 spread globally with more than 100 million confirmed infections at the end of January 2021 [1]. The main transmission route of COVID-19 is via inhalation of contaminated droplets during close person to person contact. Thus, incidence of SARS-CoV-2 in human populations is usually registered by investigation of respiratory tract samples of patients with reverse transcription quantitative (RT-q) PCR which is numerically limited, expensive and uncertain from an epidemiological point of view [2]. Additionally, the unknown relation of symptomatic and asymptomatic patients infected with SARS-CoV-2 further complicates the efforts to obtain actual overviews of virus spread in human populations. In addition to the primary transmission route of COVID-19, many studies provided evidence for fecal shedding of SARS-CoV-2 by infected patients [3], which might result in the occurrence of specific RNA in domestic wastewater. Despite different open questions (fraction of infected patients shedding SARS-CoV-2 via feces, duration of fecal virus shedding during infection, range of virus concentrations in stool samples), the transport of SARS-CoV-2 by feces to central wastewater treatment plants (WWTP) offers the possibility for a more integrated view on the regional virus dynamics in the catchment area of the WWTP by wastewater-based epidemiology (WBE) of COVID-19. The successful detection of SARS-CoV-2 up to more than 10^6^ copies/L in wastewater samples of many countries (reviewed in [4] and [5]) confirms the potential of WBE as an additional tool in the repertoire of approaches to record the course of virus incidence. However, a number of methodological aspects will influence the practical significance of data obtained from wastewater investigations, like RNA extraction in the presence of PCR inhibitors occurring in wastewater, RT-qPCR selected for detection, volume of water processed, and, not least, the efficiency of the procedure used for concentration of viruses from water. To date, different methods are utilized to enrich SARS-CoV-2 successfully from raw wastewater samples including ultrafiltration, precipitation, electronegative charged filters, and AlCl_3_ flocculation [4,5]. In addition to practical aspects of a virus enrichment method, estimation of recovery is a precondition for accuracy of future endeavors to quantify virus loads for the development of models to predict the virus circulation among humans using WBE [6].

In the present study, we evaluated two commonly used methods (precipitation with polyethylene glycol (PEG)/NaCl and ultrafiltration) to concentrate SARS-CoV-2 from untreated wastewater spiked with quantified virus-containing clinical material. With both methods, SARS-CoV-2 was previously detected in wastewater indicating their general suitability to be integrated into standard procedures for WBE of COVID-19. However, great differences of recovery rates obtained in previous studies require further evaluations of these concentration methods.

## 2. Results and Discussion

**Figure 1:**
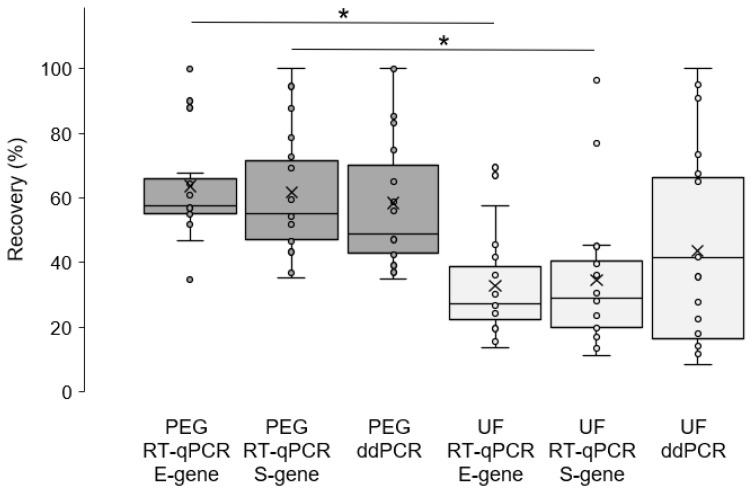
SARS-CoV-2 recovery in spiked wastewater samples using polyethylene glycol/NaCl precipitation (PEG) and ultrafiltration with Vivaspin columns (UF). RT-qPCR—real time quantitative PCR. ddPCR—droplet digital PCR. * statistical significance (*t*-test, *p* < 0.05).

The potential of WBE for an early and relatively inexpensive monitoring of SARS-CoV-2 circulation in regional human populations needs further studies. Quantification of the efficiency of the method that is selected to concentrate the viruses from wastewater plays a central role for the evaluation of monitoring data [6]. Most of previous studies used surrogate viruses, like murine hepatitis virus (MHV), to determine the enrichment efficiency of concentration methods [7]. However, structural differences between envelopes of phylogenetically-related model viruses and SARS-CoV-2 cannot be excluded and may influence the recovery after enrichment of water samples [8]. Furthermore, results depend on nucleic acid extraction and the detection approach [9]. In the present study, the calculation of efficiencies of the virus concentration methods is based on the same RNA extraction and RT-qPCR procedures as well the use of the same standard curves (Figure S1) for calculation of genome copies in all samples. Enrichment of SARS-CoV-2 in spiked wastewater with PEG precipitation resulted in mean recovery rates of 63.7% (RT-qPCR, E-gene), 61.8% (RT-qPCR, S-gene) and 59.4% (ddPCR, E-gene) (Figure 1). Differences between these rates are statistically not significant. Further details of experiments can be found in Supplementary Material (Table S1). The recoveries are in the range of results of recent studies demonstrating enrichment rates between 30% and 53% (depending on the method of nucleic acid extraction), and 44% for concentration of gamma-irradiated SARS-CoV-2 and freshly prepared MHV from untreated wastewater with PEG precipitation [7,10]. In contrast, other studies reported significantly lower mean recoveries of approximately 3% and 9% after PEG-concentration of wastewater samples seeded with surrogate virus OC43 (human betacoronavirus 1) or with SARS-CoV-2 positive clinical material [11,12]. For the interpretation of discrepancies, differences in details of the concentration methods may be important, including pH adjustment of the sample, removal of particular substances from the sample, and time and temperature of incubation of samples with PEG, respectively. Furthermore, degradation of SARS-CoV-2 by freezing/thawing and during storage of clinical material cannot be excluded and may result in partly non-infectious particles that are used to spike wastewater in the present study. Independently from the method, the recovery rate of stored clinical material containing SARS-CoV-2 from water can be influenced in comparison with intact viruses. However, this might reflect the realistic situation in wastewater systems. Despite significant RNA stability in water [13], propagation of viruses from SARS-CoV-2-positive waters was not successful [14] indicating the rapid degradation of the enveloped viruses under environmental conditions. Alternatively, spiking of water with freshly grown (intact) SARS-CoV-2 needs extensive efforts to match the biosecurity requirements which is limited to only few laboratories. However, this approach remains the gold standard for accurate quantification of virus recovery. In a further study, Ye et al. [15] also used MHV to spike wastewater and reported a mean recovery of only 5% after PEG precipitation. In this study, spiked samples were centrifuged before precipitation which might explain the low recovery as solid-bound viruses were removed. First reports suggested the importance of the association of SARS-CoV-2 with solids in wastewater systems [14,16]. Using non-enveloped echovirus 7 and mengovirus as well the enveloped porcine epidemic diarrhea virus (member of the *Coronaviridae* family) for spiking, mean efficiencies of PEG precipitation of wastewater samples of 78%, 28% and 9% were found [10,17] indicating the influence of species-specific virus characteristics on recovery.

Vivaspin columns were capable of concentrating added viruses with efficiencies of 33.0% (RT-qPCR, E-gene), 34.1% (RT-qPCR, S-gene) and 42.6% (ddPCR). Thus, recovery of SARS-CoV-2 with PEG precipitation of samples of same origin and spiked with the same virus input was significantly higher (RT-qPCR) in comparison to ultrafiltration with Vivaspin columns. Further experiments are needed to clarify if the use of other filtration columns are more effective to enrich SARS-CoV-2 from wastewater. Ahmed et al. [8] demonstrated recoveries of 28% and 56% with Centricon and Amicon columns, respectively, for concentration of wastewater samples spiked with MHV and discussed differences in the design of both devices. Additionally, adsorption of viruses to the membrane of filter columns cannot be excluded and depends on specific properties of the membrane material. Using Vivaspin 50 kDa MWCO, Trottier et al. [18] were able to detect SARS-CoV-2 in wastewater of the City of Montpellier, France, confirming the suitability of these columns in practice.

Interestingly, detection of SARS-CoV-2 by ddPCR resulted in recovery rates comparable to those obtained by RT-qPCR. ddPCR is characterized by absolute target quantification in combination with a minimal risk of inhibition of amplification [5]. In comparison with RT-qPCR (E-gene), higher concentrations of specific genome copies after detection with ddPCR in 63% of spiking material and 47% of concentrated wastewater samples were determined (Figure 1). Further investigation of greater collections of samples with naturally occurring SARS-CoV-2 in wastewater are needed to show if the use of ddPCR allows a more sensitive detection of viruses. For the establishment of an improved protocol, this includes the comparison with other targets besides the E-gene.

In addition to the advantages of precipitation (no pre-conditioning of water, possibility of concentration of higher volumes of wastewater, low costs), the time needed (around 3.5 h) and the handling efforts for the procedure are important aspects if greater numbers of samples must be processed. In comparison to ultrafiltration, co-enrichment of substances inhibiting the subsequent PCR was also described as a disadvantage of PEG precipitation [19]. In the present study, the investigation of RNA samples without additional treatment (Zymo) to remove inhibitory substances from concentrates of both enrichment procedures resulted in clear inhibition (>2 Ct-values in comparison to the negative control) of RT-qPCR in many samples. In contrast, a significant reduction of Ct-values of any sample after using the PCR inhibitor removal kit could not be found.

Further studies are necessary to determine the detection limit of the methods for analysis of SARS-CoV-2 from wastewater. With the approaches used in the present study, SARS-CoV-2 concentrations down to 1822 (mean of both RT-qPCR and ddPCR; experiment #19; Table S1) genome copies in 40 mL wastewater could be consistently tested as positive after enrichment with PEG precipitation or ultrafiltration. Additional experiments (n = 3) with lower virus concentrations between 275 and 825 copies in the spike material (addition of clinical material with higher Ct-values) demonstrated negative results with one or both PCR tests. Thus, the presence of down to approximately 4.6 × 10^4^ copies/L could be confirmed as detectable, demonstrating the need to test optimizations of the current method (e.g., by increasing the volume of processed wastewater). Depending on the method to extract nucleic acids, a recent report calculated detection limits between 1.56 and 2.34 log genome equivalents (ge)/mL (corresponding to 1452 and 8751 ge/40 mL) of after PEG precipitation of wastewater samples (200 mL) spiked with gamma-irradiated SARS-CoV-2 [11].

In conclusion, the results of the present study contribute to the further knowledge of the quantitative efficiency of methods to concentrate SARS-CoV-2 from untreated wastewater. With overall recovery rates of 61.6% vs. 36.6%, PEG/NaCl precipitation was found to be superior to ultrafiltration with Vivaspin 10 kDa MWCO columns.

## 3. Material and Methods

### 3.1. Wastewater Samples

The wastewater samples used for spiking and concentration of SARS-CoV-2 were collected from WWTP of the city of Dresden (south-east Germany) treating wastewater from nearly 650,000 inhabitants. Samples were stored at −20 °C until further processing. Immediately before spiking, water samples were thawed and centrifuged (3300× *g*, 30 min, 4 °C) to remove particular material that cause rapid clogging of filtration columns. Aliquots of non-spiked wastewater samples were tested for SARS-CoV-2 by methods described below (concentration by precipitation) and were found negative.

### 3.2. Spiking of Wastewater with SARS-CoV-2 Positive Clinical Specimens

Nasopharyngeal swabs (originated from regular SARS-CoV-2 diagnostics in the Institute of Medical Microbiology and Virology, Dresden, Germany) in 0.9% NaCl solution pretested positive for SARS-CoV-2 by a commercial RT-qPCR approach targeting the E- and S-gene (RealStar, Altona diagnostics, Hamburg, Germany) were stored at −80 °C up to 90 days before these samples were used as spike material. The samples were thawed once on ice and mixed shortly by vortexing. An aliquot (200 µL) was used for the quantification of the input virus concentration and kept on ice until RNA extraction. With further aliquots (10 to 100 µL), 80 mL of wastewater was spiked, mixed and divided in two 40 mL-samples.

### 3.3. Virus Concentration

Spiked wastewater samples (40 mL each) were concentrated by two different methods. First, the PEG (MW 8000)/NaCl precipitation was used as described [20] with slight modifications (concentration of PEG, resuspension of pellet). Briefly, 10% (*w*/*v*) PEG (Carl Roth, Karlsruhe, Germany) and 2.25% NaCl (Carl Roth) were added to the sample and mixed (head over head) at room temperature until complete solving of the additives (15–20 min). Samples were centrifuged at 4 °C and 12,000× *g* for 2 h in an Avanti HP23 centrifuge (Beckman Coulter, Brea, CA, USA). Supernatant was carefully decanted and sediment was suspended in 500 µL sterile PBS (pH 7.4; Gibco, Paisley, UK). The samples were kept on ice until further processing (within the next 30 min). Second, while the centrifugation of the first half of the sample with PEG-precipitation took place, the other half of the wastewater sample was concentrated with Vivaspin columns (MWCO 10 kDa; Sartorius, Stonehouse, UK) by centrifugation at 4 °C (3300× *g*) until the concentrate reached a volume of 700–1000 µL. The concentrated supernatant was collected and incubated on ice until RNA preparation of concentrates of both virus enrichment methods as well the spiked material.

### 3.4. RNA Extraction

Viral RNA from clinical materials as well from concentrated wastewater samples was extracted using the RNeasy kit (Qiagen, Hilden, Germany) as described by the manufacturer. Briefly, two aliquots (100 µL each) were used for preparation and eluted with 30 µL RNase-free water. Both eluents were merged and further treated with the OneStep PCR inhibitor removal kit (Zymo, Irvine, CA, USA) to reduce the concentration of substances potentially inhibiting the RT-qPCR. Treatment resulted in a final volume of 55 µL. Concentrates were immediately tested or stored at −80 °C without freezing/thawing until RT-qPCR analysis (within the next 24 h) or until droplet digital (dd) PCR (the next 8 weeks), respectively.

### 3.5. RT-qPCR

The above mentioned RT-qPCR method (RealStar) was also used to detect and quantify the concentration of SARS-CoV-2 in the RNA preparations of clinical specimens and concentrated wastewater samples. The assay was performed according to the recommendations of the manufacturer in a Quantstudio5 cycler (Thermo, Waltham, MA, USA). Each sample (5 µL) was tested in duplicate. Mean Ct-values used for quantification were based on automatic settings for threshold and baseline which were checked after RT-qPCR. In each run, a positive control (provided by the manufacturer) and a negative control (water) was included. In addition, the internal control (included in the kit) was added to all master mixes to evaluate an inhibition of PCR. For quantification of the SARS-CoV-2 genome copies, the same assay and instrument were used to establish standard curves by testing freshly prepared tenfold dilutions (in RNase-free water) of a commercial synthetic SARS-CoV-2 RNA control (Wuhan strain; Twist Bioscience, San Francisco, CA, USA). For each dilution, five parallels were tested.

### 3.6. Droplet Digital (dd)PCR

To obtain first data about the suitability of ddPCR for the detection of SARS-CoV-2 in spiked wastewater, we selected the commonly used E-gene as the target for amplification and the previously described primer/probe set [21]. ddPCR was performed on the QX200 Droplet Digital PCR System (Bio-Rad, Hercules, CA, USA), using the One-step RT-ddPCR Advanced Kit for Probes (Bio-Rad). Reactions were set up in a final volume of 20 µL following the manufacturer’s instructions, and using 4 µL of RNA. The reaction mixture was loaded into a sample well of a DG8 Cartridge (Bio-Rad) and mixed with 70 µL of droplet generator oil in the droplet generator. The droplets generated were transferred to a 96-well PCR plate (heat-sealed with a foil plate seal, Bio-Rad). Cycling conditions were 60 min at 55 °C for reverse transcription, 10 min at 95 °C for enzyme activation, followed by 40 cycles of 15 s at 95 °C for denaturation, 60 s at 58 °C for annealing/extension, and 10 min at 98 °C for enzyme deactivation, followed by an optional hold at 4 °C until droplet reading on a droplet reader. Negative (NTC, no template control) and positive (commercial synthetic SARS-CoV-2 RNA control, Wuhan strain; Twist Bioscience) controls were used in each run. ddPCR was performed without replicates. Data analysis was carried out using QuantaSoft^TM^ Software 1.7.4 (Bio-Rad). Quality controls included no amplification in NTC wells, fluorescence amplitude of positive and negative droplets, and total droplet count (>10,000). For separating negative and positive droplets, the threshold was manually set just above the cluster of negative droplets.

### 3.7. Quantification of Virus Recovery

Using the standard curves for E- and S-gene established after testing the SARS-CoV-2 RNA standard, mean Ct-values measured in all samples were converted into genome copies. Including the volume of clinical material added to untreated wastewater and the volume of water sample after concentration, a number of genome copies in the clinical material used for spiking and in the concentrated wastewater samples were compared and the rate of recovery in each experiment (n = 19) was calculated. Statistical significance of differences between mean rates obtained after RT-qPCR (E-gene and S-gene) and ddPCR (E-gene) was tested with the *t*-test (significance at *p* < 0.05).

## Data Availability

The data presented in this study can become available on request from the corresponding author.

## Supplementary Materials

The following are available online at https://www.mdpi.com/2076-0817/10/2/195/s1, Figure S1: Standard curves of RT-PCR (RealStar, Altona diagnostics) after testing tenfold dilutions (5 parallels per dilution) of SARS-CoV-2 RNA standard (Twist). Table S1. Results of concentration of untreated wastewater samples after spiking with SARS-CoV-2 positive clinical material.
