# Supplementary material for: Evaluation of Two Methods to Concentrate SARS-CoV-2 from Untreated Wastewater
Source: Pathogens. 2021 Feb 12;10(2):195. doi: 10.3390/pathogens10020195 (PMC7917696; doi:10.3390/pathogens10020195)
Supplement: Supplementary file 1 [file pathogens-10-00195-s001.pdf]

Figure S1. Standard curves of RT-PCR (RealStar, Altona diagnostics) after testing tenfold dilutions (5 parallels per dilution) of SARS-CoV-2 RNA standard (Twist Bioscience).

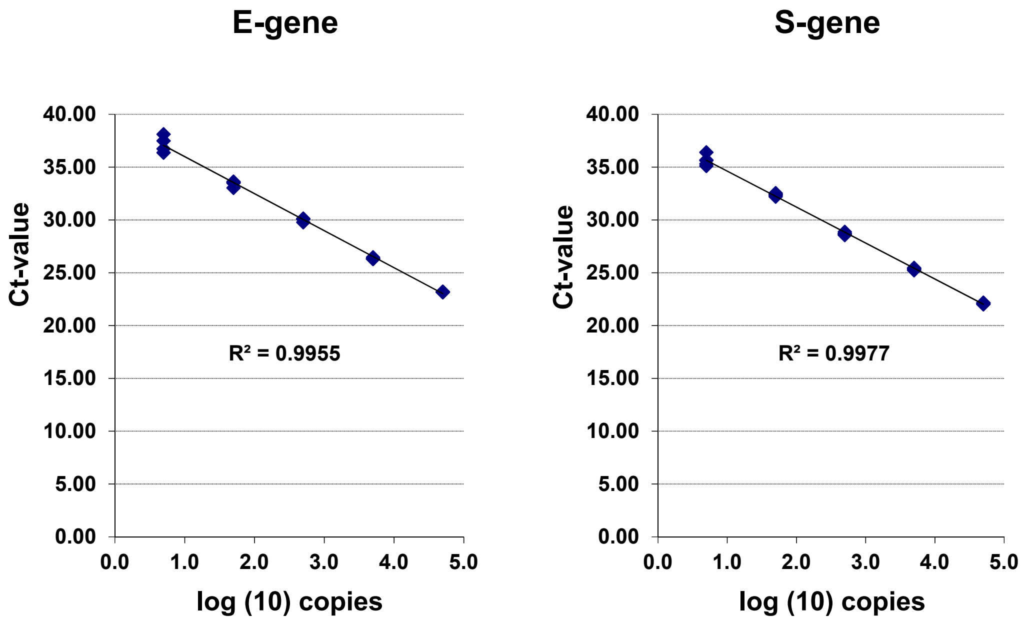

Table S1. Results of concentration of untreated wastewater samples after spiking with SARS-CoV-2 positive clinical material.

Wastewater sample	Exp. no.	Concentration method	Genome copies measured by RT-qPCR						Genome copies measured by ddPCR		
			Virus added		Virus after concentration		Recovery rate (%)		Virus added	Virus after concentration	Recovery rate (%)
			E-gene	S-gene	E-gene	S-gene	E-gene	S-gene			
1	1	Column[a]	98888	60923	27123	11515	27.4	18.9	121200	17364	14.3
		PEG[b]	98888	60923	56446	33154	57.1	54.4	121200	47432	39.1
	2	Column	39555	24369	7742	4890	19.6	20.1	48480	6353	13.1
		PEG	39555	24369	25482	14507	64.4	59.5	48480	16940	34.9
	3	Column	22295	20277	3054	2257	13.7	11.1	20120	2372	11.8
		PEG	22295	20277	13558	9479	60.8	46.7	20120	11679	58.0
	4	Column	11147	10139	3379	2842	30.3	28.0	10060	4356	43.3
		PEG	11147	10139	5859	4846	52.6	47.8	10060	4900	48.7
	5	Column	39016	35485	9621	6684	24.6	18.8	40110	7260	18.1
		PEG	39016	35485	13530	13064	34.7	36.8	40110	18879	47.1
	6	Column	27868	25346	4369	3392	15.7	13.4	28650	2468	8.6

		PEG	27868	25346	15726	13935	56.4	55.0	28650	12584	43.9
2	7	Column	11065	5041	3407	2268	30.8	45.0	10720	3812	35.6
		PEG	11065	5041	6460	4766	58.4	94.5	10720	5082	47.4
	8	Column	6916	3151	1732	743	25.0	23.6	6700	1863	27.8
		PEG	6916	3151	3586	2481	51.9	78.7	6700	2541	37.9
	9	Column	67212	36500	24282	13818	36.1	37.9	72800	49126	67.5
		PEG	67212	36500	31457	15790	46.8	43.3	72800	62073	85.3
	10	Column	20164	10950	3142	1863	15.6	17.0	21840	9317	42.7
		PEG	20164	10950	11098	4915	55.0	44.9	21840	28859	100
	11	Column	6721	3650	1629	1116	24.2	30.6	7820	5082	65.0
		PEG	6721	3650	3861	1886	57.4	51.7	7820	3812	48.7
	12	Column	12336	8291	5132	3772	41.6	45.5	12200	2753	22.6
		PEG	12336	8291	7022	5808	56.9	70.0	12200	10164	83.3
	13	Column	6168	4146	1933	1644	31.3	39.7	6100	889	14.6
		PEG	6168	4146	3546	2873	57.5	69.3	6100	2275	37.3
	14	Column	3701	2487	991	898	26.8	36.1	3660	1525	41.7
		PEG	3701	2487	2037	1373	55.0	55.2	3660	1549	42.3

3	15	Column	5202	4292	2637	1763	50.7	41.1	5800	2498	43.1
		PEG	5202	4292	3527	3761	67.8	87.6	5800	5505	94.9
	16	Column	3121	2575	2091	1979	67.0	76.8	3400	2541	74.7
		PEG	3121	2575	2745	909	87.9	35.3	3400	1906	56.1
	17	Column	2749	2106	1907	2033	69.4	96.5	5200	3812	73.3
		PEG	2749	2106	2856	2419	100	100	5200	3388	65.2
	18	Column	2748	1562	555	438	20.2	28.0	4800	4356	90.8
		PEG	2748	1562	2473	1100	90.0	70.4	4800	5929	100
	19	Column	1649	937	952	184	57.7	19.6	2880	2904	100
		PEG	1649	937	1772	681	100	72.7	2880	1694	58.8
Mean recovery ± SD		Column					33.0 ± 16.8	34.1 ± 21.6			42.6 ± 28.4
		PEG					63.7 ± 17.8	61.8 ± 18.9			59.4 ± 22.2

[a] filtration by Vivaspin columns

[b] polyethylene glycol/NaCl precipitation
